# Gating of memory encoding of time-delayed cross-frequency MEG networks revealed by graph filtration based on persistent homology

**DOI:** 10.1038/srep41592

**Published:** 2017-02-07

**Authors:** Jarang Hahm, Hyekyoung Lee, Hyojin Park, Eunjoo Kang, Yu Kyeong Kim, Chun Kee Chung, Hyejin Kang, Dong Soo Lee

**Affiliations:** 1 Department of Nuclear Medicine, Seoul National University College of Medicine, Seoul, 110-744, Korea; 2 Institute of Radiation Medicine, Medical Research Center, Seoul National University, Seoul, 110-744, Korea; 3 Interdisciplinary Program in Cognitive Science, Seoul National University, Seoul, 151-742, Korea; 4 Department of Psychology, Kangwon National University, Chuncheon-si, 200-701, Korea; 5 Data Science for Knowledge Creation Research Center, Seoul National University, Seoul, 151-742, Korea; 6 Department of Nuclear Medicine, Seoul National University Boramae Medical Center, 156-707, Seoul, Korea; 7 MEG Center, Department of Neurosurgery, Seoul National University College of Medicine, Seoul, 110-744, Korea; 8 Department of Molecular Medicine and Biopharmaceutical Sciences, Graduate School of Convergence Science and Technology, and College of Medicine or College of Pharmacy, Seoul National University, Seoul, 110-744, Korea; 9 Institute of Neuroscience and Psychology, University of Glasgow, Glasgow, United Kingdom

## Abstract

To explain gating of memory encoding, magnetoencephalography (MEG) was analyzed over multi-regional network of negative correlations between alpha band power during cue (cue-alpha) and gamma band power during item presentation (item-gamma) in Remember (R) and No-remember (NR) condition. Persistent homology with graph filtration on alpha-gamma correlation disclosed topological invariants to explain memory gating. Instruction compliance (R-hits minus NR-hits) was significantly related to negative coupling between the left superior occipital (cue-alpha) and the left dorsolateral superior frontal gyri (item-gamma) on permutation test, where the coupling was stronger in R than NR. In good memory performers (R-hits minus false alarm), the coupling was stronger in R than NR between the right posterior cingulate (cue-alpha) and the left fusiform gyri (item-gamma). Gating of memory encoding was dictated by inter-regional negative alpha-gamma coupling. Our graph filtration over MEG network revealed these inter-regional time-delayed cross-frequency connectivity serve gating of memory encoding.

Cognitive processes have been suggested that it arise from changes in interactions of regional oscillatory activity in a large-scale brain network^1^. Capturing a network configuration of brain connectivity of these oscillations on the temporo-spectral domain will inform us of ongoing cognitive processes. In this study, we examined the regulation of long-term memory encoding by finding the relevant network or connectivity of alpha-gamma power coupling from cueing to encoding interval across distributed brain areas.

Gating of memory encoding is a process to allow encoding relevant information while blocking irrelevant one. It is supposed to be an essential process considering we encounter innumerable information in our daily life. Most studies on neural correlates of gating memory encoding have focused on addressing the question of which oscillatory power or a specific region is involved in this process^2^,^3^,^4^,^5^,^6^,^7^. Recently, one MEG study found alpha power increase for blocking irrelevant information while gamma power increase for encoding relevant one during encoding of long-term memory^7^. Furthermore, the study suggested the negative relationship between alpha and gamma power cross-frequency coupling for regulating memory encoding by focusing on the posterior region. From a view of aforementioned large-scale network perspective, we hypothesized that distributed large-scale network or connectivity may contribute to gating memory encoding.

The present study aimed to expand the previous MEG study^7^ by estimating a large-scale brain network including inter-regional distributed connectivity which serves for gating of long-term memory encoding. Using MEG data of Remember (R) and No-Remember (NR) condition of the encoding session for the cued long-term memory task (Fig. 1a), we model spatially distributed networks by adopting the time-delayed cross-frequency relationships between alpha band power during cue presentation (cue-alpha) and gamma band power during item presentation (item-gamma) (Fig. 1b). To tackle the complexity inherent in the analysis of the coupling with variable strengths between the different spectrums of different areas, we adopted the persistent homology framework using graph filtration^8^. Specifically, we adopted bipartite graph filtration to sort and compare the spatially distributed time-delayed cross-frequency connectivity data.

Graph filtration based on the persistent homology extracts the most relevant topological invariant out of big and complex data^9^. Interpretation of networks by graph filtration and persistent homology has had an advantage over interpretation of binary graphs made by thresholding the weights, as graph filtration circumvents the problem of arbitrariness of selecting the threshold on edge weights. Thresholding has long been plagued by the subjectivity due to this arbitrariness and the ensuing probable loss of the information contained in the weighted graphs^8^,^10^.

MEG data enable comprehensive spatio-temporo-spectral observation of brain oscillations and thus render immense challenges associated with escalating complexity due to their multi-dimensionalities. Here at the beginning of an exploration of the huge MEG data by choosing alpha-gamma cross-frequency with time delay from cue and item periods, we reduced temporal-spectral multi-dimension to one dimension and detoured this problem by treating the resulting data as disjoint sets of many brain regions in the form of bipartite graph (Fig. 2). We then applied bipartite graph filtration and the differences of filtered networks between R and NR condition were examined using a tailored statistical test. As the bipartite graphs were filtered by varying the thresholds, threshold-free interpretation became feasible and so did the comparison of time-delayed cross-frequency coupling and non-time-delayed within-frequency coupling for the distributed cortical regions of the brain. Among the multi-level topological invariants, Betti-0, the number of connected components was attended. Barcodes and dendrogram were shown for each condition and their equivalent single linkage distance matrices (SLMs) were analyzed. We looked for which regional pairs of inter-regional coupling of time-delayed cross-frequency alpha-gamma powers were related to the differences between R and NR condition and which regional pairs were predictive of good or poor memory performances of the individual subjects. As we focused on the negative correlation between alpha and gamma, the probable regional adjacency effect of positive correlation could be easily disregarded.

Exploring the threshold-free characteristics of distributed functional brain networks on MEG, we could find that the inter-regional time-delayed cross-frequency coupling was engaged in gating of long-term memory encoding in R and NR task condition. We hypothesized that task instructions for either memory encoding or its blocking were treated in the brain by the gating processes of cross-temporo-spectral coupling across distributed regions and subsequently its strength would further predict the individual subjects’ performances of memory during R and NR condition.

## Results

### Behavioral performances

R-hits ratio (57% ± 3.2%) was significantly greater than NR-hits ratio (33% ± 2.2%) (*P* < 0.001), showing participants remember more items following the R cue than items following the NR cue as they were instructed. Both R-hit and NR-hit ratios were significantly greater than false alarm ratio (24% ± 2.0%) (Both were *P* < 0.001). This result indicated that items shown in the encoding session were less likely to be recognized by guessing. See ref. 7 for more details.

### Barcodes and dendrograms of time-delayed cross-frequency networks in Remember and No-Remember condition

The aim of the present study was to investigate pre-stimulus alpha and post-stimulus gamma power modulation in a large-scale network for each Remember and No-Remember condition. The relationship between cue-alpha and item-gamma can be modeled by the bipartite graph (Fig. 2b), which is composed of the nodes that are partitioned into two subsets and the edges that are only allowed to connect two nodes in different subsets. Thus, the bipartite graph yields an asymmetrical or non-autologous network (Fig. 2c). This is because the nodes of each row and column in the correlation matrix are from different sets, namely, different frequency (alpha and gamma) and different time (cue and item) domains in the same brain region. In the present study, different set indicated different time and frequency (i.e., cue-alpha and item-gamma). If there is a single set of nodes and their correlation matrix is estimated, the edge at (*i, j*) (*a*_*ij*_) and at (*j, i*) (*a*_*ij*_) is the same (*a*_*ij*_ = *a*_*ji*_) since two target nodes for each edge are the same: (*x*_*i*_, *x*_*j*_) = (*x*_*j*_, *x*_*i*_). However, for the bipartite graph, *a*_*ij*_ ≠ *a*_*ji*_ because nodes linking by each edge are totally different: (*x*_*i*_,*y*_*j*_) ≠ (*x*_*j*_,*y*_*i*_) (Fig. 2d). In other words, the coupling strength between cue-alpha power at region A and item-gamma power at region B is not the same with the strength between item-gamma power at region and cue-alpha power at region B. In addition, the network included inter- and intra-regional connectivity as shown in the matrix form (Fig. 2e). In this study, we assigned two subsets of nodes to cue-alpha and item-gamma and examined how the connected structure of bipartite network is changed by interaction between cue-alpha and item-gamma based on the graph filtration. In this case, SLMs made by bipartite graph filtration could not be transformed to barcodes due to the theoretical reasons^11^ and also the practical reasons (2D invariant). The graph filtration was originally defined for the autologous (using the same variables) brain network analysis in PET^8^, MRI^12^, and fMRI^13^. Here, we extended this method to analyze the bipartite graph filtration of negative correlation between cue-alpha and item-gamma. To investigate the global pattern of time-delayed cross-frequency coupling engaging in gating sensory information or memory encoding, the barcode was calculated. The barcode is the visualization tool for the change of connected structure during the graph filtration by keeping tracking the number of connected component (i.e., the 0th Betti). For the bipartite network, barcodes were separately produced for each disjoint set of nodes (i.e., cue-alpha and item-gamma) from time-delayed cross-frequency network. Finally, the bipartite graph filtration yielded two barcodes, and we called them cue-alpha barcode of time-delayed cross-frequency network and the item-gamma barcode of time-delayed cross-frequency network, respectively.

Compared with the barcode of cue-alpha of non-time-delayed within-frequency network, the barcode of cue-alpha of time-delayed cross-frequency network showed shorter distance among regions in both Remember (*P* < 0.0001) and No-Remember condition (*P* < 0.0001) (Fig. 3a). Item-gamma barcode of time-delayed cross-frequency network also showed shorter distance among regions than that of non-time-delayed within-frequency network in both Remember (*P* < 0.0001) and No-Remember condition (*P* < 0.0001) (Fig. 3b). The dendrogram displayed in bottom of Fig. 3a and b showed again the differences between time-delayed cross-frequency networks and non-time-delayed within-frequency networks in each cue-alpha and item-gamma, respectively.

Cue-alpha of time-delayed cross-frequency networks did not show significant difference between R and NR condition (*P* = 0.14, permutation test) (Fig. 3c, Left) while item-gamma of time-delayed cross-frequency networks showed significantly shorter distance among regions in R than in NR condition (*P* = 0.01, permutation test) (Fig. 3d, Left). The differences of barcodes (defined by the difference of the number of connected components (*β*_0_) whose absolute value is the maximum difference over the filtration values, which called diff_max_) for cue-alpha of time-delayed cross-frequency network between R and NR condition were not significantly correlated with instruction compliances (Fig. 3c, Right), while diff_max_ for item-gamma of time-delayed cross-frequency network were significantly correlated with instruction compliances (*r* = −0.50, *P* = 0.02) (Fig. 3d, Right).

### Instruction compliance was related to interregional negative coupling between cue-alpha and item-gamma

The connectivity of SLM between *i-*th node of cue-alpha (*x*_*i*_) and *j-*th node of item-gamma (*y*_*j*_) 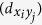 were compared between Remember and No-Remember condition (i.e., 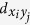 of R minus 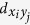 of NR) (Fig. 4a). The pairs of the left superior occipital gyrus (SOG) of cue-alpha and the left dorsolateral part of the SFG (SFG.dl) of item-gamma, the right middle temporal gyrus (MTG) of cue-alpha and the left orbital part of the middle frontal gyrus (MFG.orb) of item-gamma showed stronger negative coupling in Remember than No-Remember condition (*P* < 0.0005, permutation). The other five regional pairs also showed stronger negative coupling in Remember than No-Remember condition between the following five regions of cue-alpha (namely, the right opercular inferior frontal gyrus (IFG.op), the left cuneus, the left superior part of temporal pole (TP.STG), the left supramarginal gyrus (SMG), and the left orbital superior frontal gyrus (SFG.orb)) and the left insula of item-gamma (Fig. 4b, Table 1).

Among the seven coupled pairs, 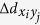 between Remember and No-Remember condition was correlated significantly with instruction compliances (i.e., standard *d* prime of R-hits minus NR-hits) in the pair of the left SOG of cue-alpha and the left SFG.dl of item-gamma (*r* = −0.58, *P* = 0.004, Bonferroni-corrected *P* = 0.028) (Fig. 4c). It indicated that stronger negative coupling in R condition between the left SOG and the left SFG.dl represented higher compliance.

### Memory performance was related to interregional negative coupling between cue-alpha and item-gamma

We compared the barcodes between good (>1.0 of standard *d*-prime of R-hits minus false alarms) and poor (<1.0 of standard *d*-prime of R-hits minus false alarms) performers of the difference between conditions (Remember vs. No-Remember) for each cue-alpha and item-gamma barcode of time-delayed cross-frequency network. The difference between good and poor performers in the difference (Remember vs. No-Remember) for the cue-alpha barcodes was not significant (*P* = 0.20, permutation test), while that for the item-gamma barcodes was significant (*P* = 0.03, permutation test).

Among the above seven coupled pairs found by SLD comparison between task conditions (Remember vs. No-Remember), 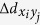, tended to be correlated with memory performances (standard *d* prime of R-hits minus false alarms) in the pair of the left SOG of cue-alpha and the left SFG.dl of item-gamma (*r* = −0.43, *P* = 0.04, Bonferroni-corrected *P*-value = 0.28). Moreover, thus, we compared good and poor performers to find the interregional pairs involved in memory performance (Fig. 5a). The differences of 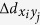 (Remember vs. No-Remember) between good and poor performers were significant in the pair of the right posterior cingulate cortex of cue-alpha and the left fusiform gyrus of item-gamma (*P* < 0.0005, permutation) (Fig. 5b). 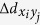 (Remember vs. No-Remember) was correlated significantly with memory performance in the pair of the right posterior cingulate cortex of cue-alpha and the left fusiform gyrus of item-gamma (*r* = −0.64, *P* = 0.001). Shorter distance in Remember condition was related to higher memory performance, which meant that stronger negative coupling in Remember condition between the right posterior cingulate and the left fusiform could predict memory performances of individuals (Fig. 5c).

## Discussion

In the present study, to understand the regulation of brain oscillation correlates of gating of memory encoding, interregional correlation of time-delayed cross-frequency negative coupling of alpha-gamma powers of various brain areas were examined. Intra- and inter-regional negative correlations were graph-filtered based on persistent homology framework, without setting any threshold eventually to obviate the arbitrariness of choosing threshold. Bipartite graph filtration disclosed that there was the distributed interregional coupling not appearing on within-frequency coupling, which was displayed in the barcodes and dendrogram of time-delayed cross-frequency coupling of distributed brain areas. Among these regional pairs of inter-regional negative coupling, instruction compliance (R-hits minus NR-hits) was significantly associated with the coupling between the left superior occipital and the left dorsolateral superior frontal gyri. The inter-regional alpha-gamma coupling of these left occipital-frontal areas showed a tendency of association with memory performance but failed to reach a statistical significance. To find the connectivity associated with memory performance, we divided participants into good and poor performers and made a contrast between groups. The connectivity showing significance difference between groups explained variability of memory performance (R-hits minus false alarm) in the pair of the right posterior cingulate cortex of cue-alpha and the left fusiform gyrus of item-gamma. Our finding suggested that inter-regional and distributed brain connectivity support gating of encoding process by facilitating remembering or by blocking no-remembering information. In particular, occipital-frontal alpha-gamma negative coupling is supposed to support instruction compliance, and cingulate-fusiform alpha-gamma negative coupling is associated with the eventual success of remembering, which would serve the memory performance of the individuals.

Uniquely in this investigation, inter-regional time-delayed cross-frequency negative coupling was modeled in the framework of persistent homology which avoids the problems the thresholding could lead to. By overcoming the subjectivity and ambiguity of setting arbitrary threshold, we could confidently show that the time-delayed cross-frequency negative couplings of certain regional pairs are in charge of instruction compliance and memory performance respectively. And thus, we maintain that gating of memory encoding showing up in MEG brain oscillation data during R and NR conditions can be delineated with our methods of graph filtration for inter-regional negative coupling measure.

The global comparison of time-delayed cross-frequency negative coupling between R and NR condition disclosed differentially-connected regional pairs serving for instruction compliance. As expected, in contrast to the improbable within-frequency negative coupling of either cue-alpha or item-gamma, time-delayed cross-frequency negative coupling was definitely present between various brain regional pairs and bipartite graph filtration and its barcode presentation clearly showed the difference of the coupling between R and NR condition. As the barcode is the shuffled dendrogram and dendrogram could not easily be compared statistically between conditions, we referred to SLMs when we tried to find the inter-regional pairs to explain the difference of brain oscillation correlates in R and NR condition. Statistical comparison between R and NR SLMs using permutation methods, we could discover seven regional pairs, with more conservative criteria (*P* < 0.0005). However, instruction compliance was significantly correlated with SLD differences between R and NR only in the pair of the left superior occipital gyrus and the left dorsolateral part of the superior frontal gyrus. Alpha power of the left superior occipital gyrus of cue period was negatively correlated with gamma power of the dorsolateral part of the superior frontal gyrus of item period, which was stronger in R condition.

The difference of barcodes between R and NR condition was negatively correlated with instruction compliance (Fig. 3d, Right). The close coupling among regions reflected by barcodes of graph filtration of R condition would have meant the higher compliance to R instruction (higher hit ratio of R). Or in the other way round, the later merging of barcodes of NR would have meant the successful blocking of memory and thus higher compliance to NR instruction (lower hit ratio of NR). Memory is encouraged at instruction of R and if the subjects comply with instruction of R then correct hit ratio (i.e., R-hits) is high and memory blocking is encouraged at instruction of NR and if the subjects comply with instruction of NR then false hit ratio (i.e., NR-hits) becomes low. Both high R-hits and low NR-hits will yield higher value of instruction compliance. Note that the relationship between instruction compliance and barcode difference between conditions was observed at item-gamma of time-delayed cross-frequency network but not at cue-alpha of time-delayed cross-frequency network. Now we could say that the interregional time-delayed cross-frequency negative coupling of brain regional pairs was definitely different between R and NR condition according to the instruction compliances in particular.

On the temporal axis, cue precedes item and occipital alpha during cue initiates and frontal gamma during item follows. Occipital might be the area of the initiation of memory or memory blocking, i.e. gating of memory encoding, while frontal might be the area of completion of instruction response. Instruction compliance might be accomplished at item presentation at the superior frontal area associated with various brain areas. Interestingly, only the left occipital-frontal pair was found to be responsible for instruction compliance. The tighter interregional negative coupling between the left occipital alpha and the left frontal gamma in Remember compared with No-Remember condition was predictive of higher instruction compliance.

Occipital alpha power has been reported to perform top-down modulation for subsequent memory performance^14^ and the superior frontal gyrus was reported to be involved in memory tasks^15^. Taken together, the top-down control reflected by occipital alpha power may exert on memory encoding by frontal gamma power to encode Remember items while blocking No-Remember items at the encoding stage. These findings are compatible with the notion that gating of visual processing depends on posterior alpha activity^16^,^17^.

Comparison of the barcode between good and poor memory performers revealed that the difference between good and poor performers resided in the item-gamma of time-delayed cross-frequency negative coupling but not in the cue-alpha coupling. Among the seven interregional pairs of tighter negative coupling in Remember than No-Remember condition, previous interregional pair between the left occipital and the left frontal region tended to show relation with memory performance, but not statistically significant (corrected *P*-value > 0.05). Rather, the comparison of good and poor performer groups revealed another interregional pair of the right posterior cingulate and the left fusiform, which showed significant relationship with memory performance. In this study using MEG data and bipartite time-delayed cross-frequency coupling, we propose that the tighter negative coupling between cingulate alpha and fusiform gamma in Remember condition is associated with higher memory performance. However, we do not imply either that this inter-regional time-delayed cross-frequency coupling represent solely the higher performance of memory encoding or alternatively that this coupling represent the lower interfering effect of intended blocking of memory encoding.

Memory has been suggested to emerge through distributed regional integration^18^,^19^,^20^. In particular, posterior cingulate cortex is not only related to internally directed thought as a part of default mode network, but also connected to the cognitive control^21^,^22^. Gamma power in fusiform gyrus is modulated by attention^23^ and the fusiform gyrus shows greater activation during successful encoding of pictorial item for the long-term memory^24^. Based on these findings, our results suggest that cognitive control by posterior alpha band power influences the encoding process by occipital gamma band power, which enable to encode Remember items and in turn recognize new items as a novel item correctly.

In fact, the current study did not directly measure the causal influence of alpha band power to gamma band power. For more support for the causal influence, a further study using the transcranial magnetic stimulus (TMS) will be required in the setting of interfering alpha (10 Hz) band power of the left SOG while measuring the gamma band (80 Hz) power of the left dl.SFG. Or based on successive occurrence of two functional states, causality analysis will warrant the gating mechanism of the pre-stimulus alpha band power to the post-stimulus gamma band power.

In the current study, we expanded the previous studies attending to intra-regional time-delayed cross-frequency negative coupling to that of inter-regional coupling. The information in MEG data depicting brain oscillations and their decomposition to the time-frequency domains yields huge complex data which usually deter any easy inter-regional analysis. In addition, the coupling (or connection) between multi-regional time-frequency power data are varied in their strength, and thus if we set the arbitrary threshold, we may lose information in MEG data and objectivity of data processing. Arbitrary setting of the thresholds will make interpretation subjective, ambiguous and biased. Successful application of the perspectives of persistent homology to MEG time-frequency data was realized in this study by the application of graph filtration. However, we faced another problem of filtering the graph made of time-delayed cross-frequency coupling of all the regional pairs over the brain. This problem was solved by modeling the bipartite network.

The advantage of our methods can be outlined as follows. First, as described above, this method is threshold-free, avoiding any arbitrary selection of threshold or possibility of information loss. Moreover, it provided the invariant measure of network, such as barcode and single linkage dendrogram. In our previous report, we found that barcode is shuffled form of dendrogram, both of which are equivalent with SLM (See ref. 8). SLMs could be analyzed to disclose where the interregional pairs of differential coupling are between R and NR condition or between good and poor performers.

Second, this method is appropriate for an exploratory study^25^. We did not assume any global or regional coupling in particular a priori. Among the several pairs showing difference between groups, we did correlation analysis with instruction compliance and memory performance. Bonferroni-correction enabled us to conservatively interpret our exploratory study results, some of which are post-hoc studies. To compare the SLMs of different groups, we used permutation methods with sufficient iteration. As we did not assume any distribution, we could also collect the meaningful interpretation with confidence.

Third, using this method, one can elucidate the basic structure of biological data. The method could disclose similar topological feature between the spontaneous neuronal population activity and the activity driven by natural image sequence in primary visual cortex^25^. The finding was consistent with the previous reports in the literature, suggesting that the topological analysis help to uncover the underlying structure of neuronal activity. Topological core structures of brain networks were found in human pathology^8^,^12^ and in rodent model^26^,^27^, implying that the invariant features of the brain acquired with graph filtration are the core of differences in the brain network pathologies.

Information flow in the brain is considered to be processed by interactions among distributed cortical modules covering large-scale regions^28^,^29^ and covering spatial-temporal-spectral dimension^1^. In this sense, the functional brain network needs to be modeled by considering multi-dimensional interactions among multiple distributed areas of the brain. In this sense, the present study modeled a multi-dimensional brain network on MEG source activity, which will be the first step toward a multi-dimensional brain network modeling. Among the time-frequency-regional combination, we fixed time-delayed cross-frequency data to one dimension, i.e., cue-alpha and item-gamma coupling, and varied the space to multi-regional time-delayed cross-frequency data. To model the brain network using electrophysiological studies, there are several methods including seed-based approach^30^, independent component analysis^31^, and all-to-all statistical dependency calculation on source level^32^,^33^,^34^,^35^. In the present study, we used the bipartite graph filtration to estimate interregional coupling or ensuing network in terms of persistent homology. Brain networks were successfully modeled so that global and local properties of these functional network could be delineated even to explain behavioral correlates such as instruction compliance and memory performance.

In conclusion, gating over long-term memory encoding is operated by ongoing configuration of a large scale multi-dimensional brain network. In particular, gating the left occipital alpha activity to the left superior frontal gamma activity helps better instruction compliance. In addition, gating the right posterior cingulate alpha activity to the following left fusiform gamma activity is predictive of memory performance. Integrating these findings, regulation of memory encoding is subserved by interregional cross-frequency-time network between posterior alpha activity and subsequent distant gamma activity. Considering functional brain networks span multiple dimensions, graph filtration based on persistent homology framework enables multi-dimensional analysis of inter-regional temporal-spectral association of brain oscillations and exploratory studies are warranted for complex multi-dimensional MEG data.

## Materials and Methods

We employed the previously reported data^7^. The procedure from preprocessing to source localization was the same as our previous research. The difference from the previous study is that the current study modeled a spatially-distributed brain network of time-delayed cross-frequency coupling of cue-alpha and item-gamma using the graph filtration method. And we tried to find the distributed spatial network using a permutation method. Finally we searched for the connectivity explaining instruction compliance and memory performance. In this section, at first we briefly described a procedure from preprocessing to source localization (See ref. 7 for more details), and then network analysis in detail.

### Participants

Twenty-three healthy subject data (11 males and 12 females, mean age of 24.8 ± 3.1) were included for analysis. All of the participants were right-handed and had normal or corrected-to-normal vision. None of the participants had any history of developmental, psychological, or neurological disorders. All experiments were performed in accordance with ethical guidelines and regulations that have their origin from the Declaration of Helsinki. This study was approved by the Institutional Review Board (IRB) at Seoul National University Hospital (IRB No. C-1007-156-325). All the participants completed the written informed consent before the experiment and received monetary compensation for their participation.

### Experimental paradigm and procedure

**Figure 1:**
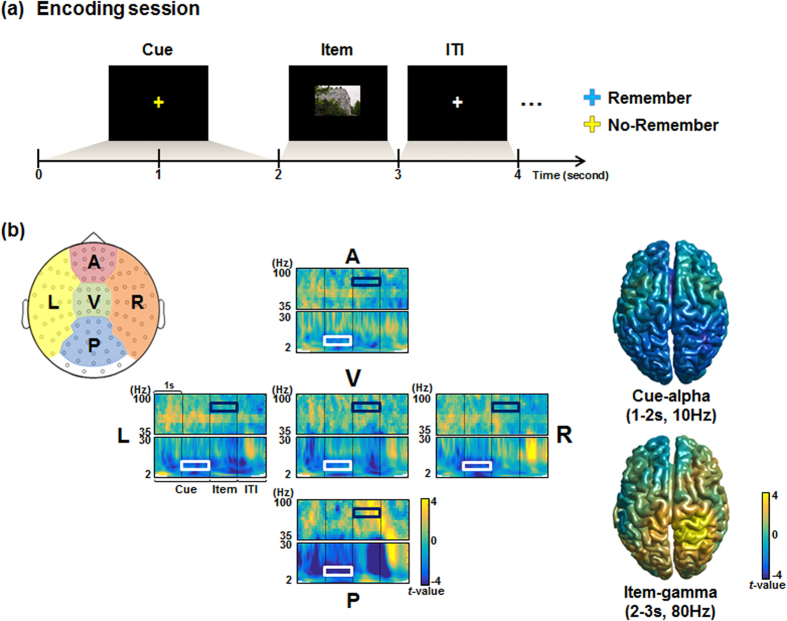
A cued long-term memory paradigm and time-frequency analysis of power. (**a**) The cued long-term memory paradigm. Only the encoding session was presented in the figure (See Materials and Methods section for the recognition session). A cue was presented prior to a pictorial item and the cue instructed the participants by its color to remember (Remember condition) or not to remember (No-Remember condition) the following pictorial item. (**b**) Time-frequency analysis of power was performed for each sensor and the power was averaged within each area as depicted in the topoplot on the left side (A: anterior, P: posterior, L: left, R: right, V: vertex region). The comparisons of power between Remember and No-Remember condition showed higher power in alpha band, but lower in gamma band in No-Remember compared with Remember condition. Among the statistically significant (*P* < 0.05) power differences between conditions, 10 Hz during cue presentation (cue-alpha) and 80 Hz during item presentation (item-gamma) were chosen and indicated with boxes (white: cue-alpha, black: item-gamma). Their source spectral power was shown on the right. ITI: inter-trial interval.

**Figure 2:**
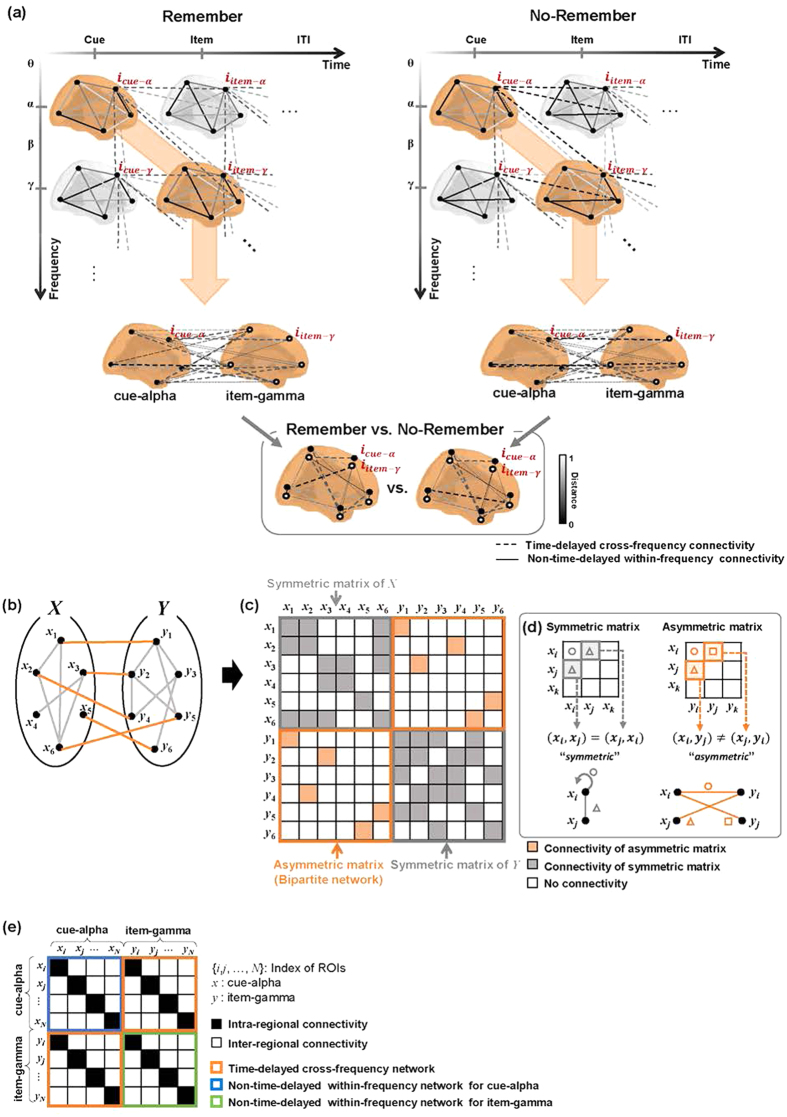
Illustration of analysis of the present study. (**a**) Among many coupling possibilities, we focused on interactions between cue-alpha (alpha power during cue presentation) and item-gamma (gamma power during item presentation) (orange brains). This time-delayed cross-frequency negative coupling was finally compared between conditions (Remember vs. No-Remember). Networks derived from within-frequency cross-time or within-time cross-frequency coupling were simply not chosen for further analysis. (**b**) The selected network of (**a**) is modeled by a bipartite graph, which consists of two disjoint sets of nodes and edges linking the nodes. Assume there are two sets of nodes, 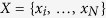 and 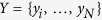, and their relationship (edges; orange lines) when *X* and *Y* indicates different properties of the same sources ({*i*, …, *N*}). To address its characteristics, we additionally illustrated the typical type of a network with single set of nodes and edges (gray lines). (**c**) Once a graph is transformed into a matrix form for analysis and display, there is an asymmetric matrix (orange box) for bipartite graph, while a symmetric matrix (gray box) for each *X* and *Y* network, respectively. Note that two asymmetric matrices are the same when transpose either of the two. (**d**) The asymmetric matrix is produced due to the fact that each row and column of the matrix is aligned by different properties (*X* and *Y*) while nodal index is the same. For the typical network, two edges at (*i, j*) and at (*j, i*) link the same index and property of nodes 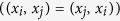, yielding a symmetric matrix. However in the bipartite graph, they links the same index but different property of nodes: 
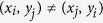
, leading to an asymmetric matrix. (**e**) In the present study, each set of nodes (*X* and *Y*) indicates cue-alpha and item-gamma and nodal indices (*i*, …, *N*) are ROI labels. The bipartite network (orange box) represents time-delayed cross-frequency network including edges within the single region (intra-regional connectivity; black) and between different regions (inter-regional connectivity; white). The properties of time-delayed cross-frequency network are examined by being compared to two non-time-delayed within-frequency networks (for cue-alpha: blue; for item-gamma: green).

**Figure 3:**
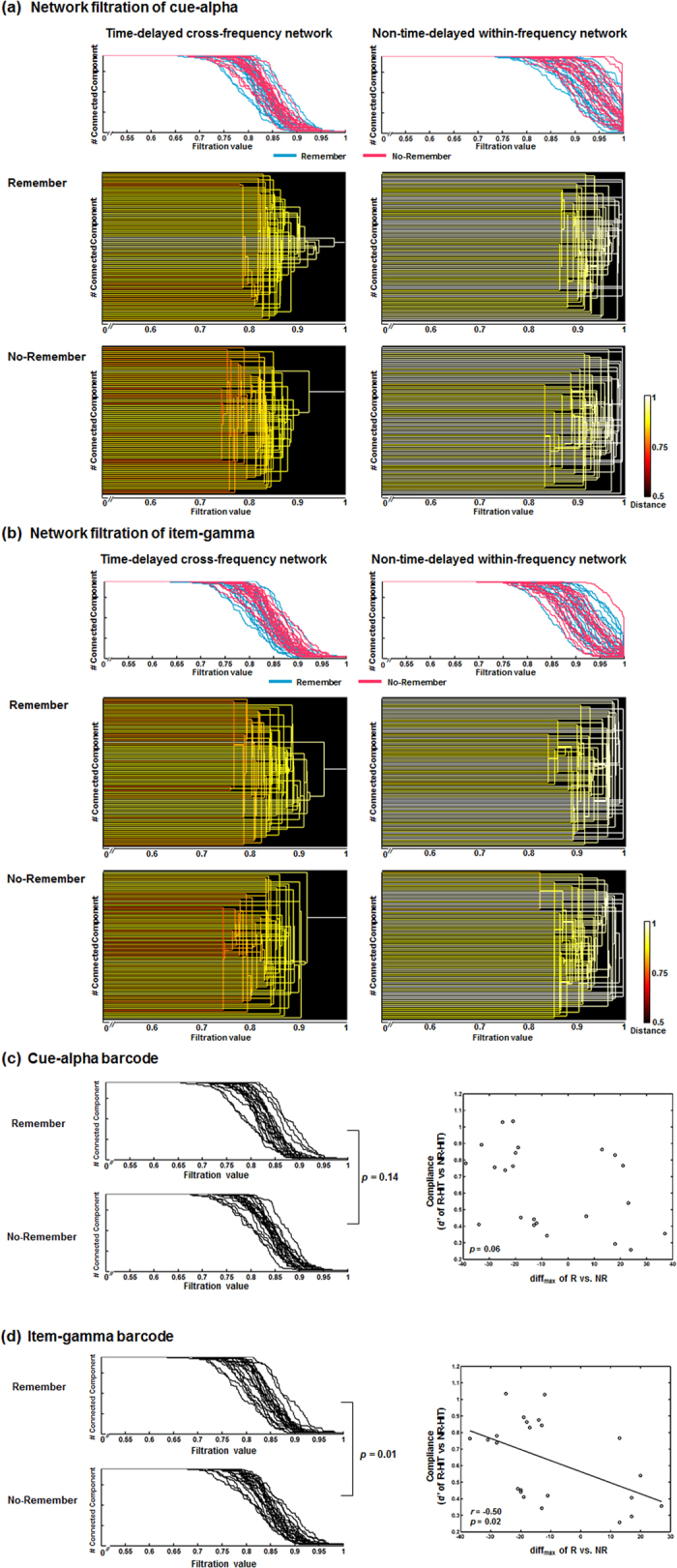
Comparisons of the barcodes and display of the dendrogram. Bipartite filtered graphs of the time-delayed cross-frequency network yielded barcodes and dendrogram for each set of nodes, cue-alpha and item-gamma. (**a**) For the cue-alpha of the time-delayed cross-frequency network, the barcodes for all subjects were shown on the top left, and blue ones were from Remember condition and red ones are from No-Remember condition. A dendrogram of a single subject (the first subject) was shown on the bottom left as an example. For comparison, the cue-alpha of the non-time-delayed within-frequency network (i.e., cue-alpha-to-cue-alpha network) with negative coupling was displayed for the barcodes and a dendrogram on the right sides. (**b**) For the cue-alpha of the time-delayed cross-frequency network, their barcodes and a dendrogram were shown on the left. Barcodes and a dendrogram of the non-time-delayed within-frequency network for item-gamma (i.e., item-gamma-to-item-gamma network) were shown on the right side. (**c**) For cue-alpha of the time-delayed cross-frequency network, barcodes of R condition did not differ significantly from those of NR condition. The differences of barcodes between conditions (R vs. NR) for the cue-alpha of the time-delayed cross-frequency network in individuals were not correlated with instruction compliance (*d*’ of R-hits minus NR-hits). (**d**) For item-gamma of the time-delayed cross-frequency network, the barcodes of the R condition was significantly different from that of NR condition, and the differences between conditions were significantly correlated with instruction compliance (*r* = −0.50, *P* = 0.02).

**Figure 4:**
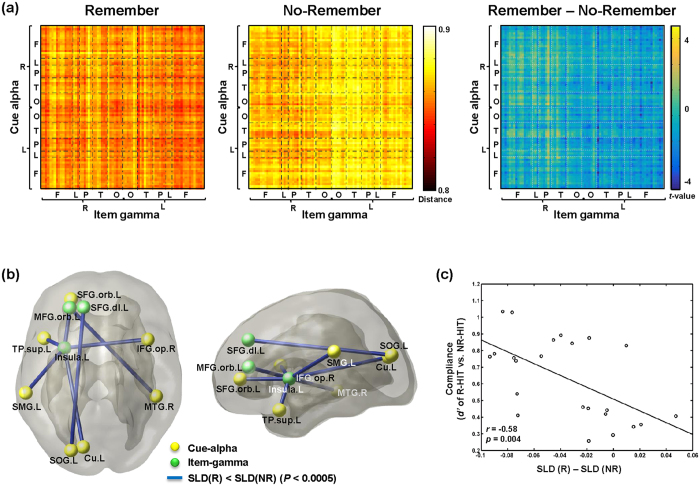
Comparisons of the single linkage distance between Remember and No-Remember condition. (**a**) The average of single linkage matrix across subjects was shown for each condition and difference between conditions. (**b**) The regional pairs showing significant differences of single linkage distances (SLD) between R and NR condition were shown on 3D brain display (*P* < 0.0005). From the left, horizontal and sagittal views are shown. (**c**) For the regional pair of the left superior occipital gyrus (SOG) of cue-alpha and the left superior frontal gyrus (SFG) of item-gamma, differences in SLDs were significantly correlated with individuals’ instruction compliance (*d*’ of R-hits minus NR-hits) (*r* = −0.58, *P* = 0.004).

**Figure 5:**
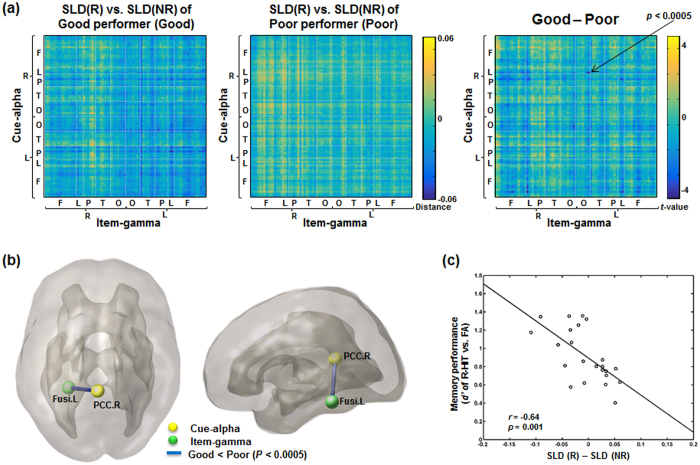
Comparisons of the single linkage distance between good and poor performers. (**a**) Single linkage distances (SLDs) showed differences between conditions (Remember vs. No-Remember) of single linkage distances of good performers (>1.0 of standard *d*-prime of R-hits minus false alarms) (left) and of poor performers ( < 1.0 of standard *d*-prime of R-hits minus false alarms) (middle), and their differences between good and poor performers (right) were shown. (**b**) The regional pairs showing significant differences between good and poor performers in the difference of SLDs between conditions (Remember vs. No-Remember) were shown on 3D brain display (*P* < 0.0005). Negative coupling between the right posterior cingulate cortex of cue-alpha and the left fusiform gyrus of item-gamma showed significant difference. (**c**) For the regional pair of the right posterior cingulate gyrus of cue-alpha and the left fusiform gyrus of item-gamma, differences in SLDs were significantly correlated with memory performance (*d*’ of R-hits minus false alarms) (*r* = −0.64, *P* = 0.001).

A cued long-term memory paradigm was employed. In the encoding session, a trial consisted of two phase: cue and item presentations. After presentation of cue for 2 s, item was presented for 1 s, followed by inter-trial interval (ITI) for 1 s (Fig. 1a). Subjects were instructed either to remember (R condition) or not to remember (NR condition) the following picture item according to a color (i.e., yellow or blue) of the prior fixation cross. The item was real-life photographs of landscapes or buildings. The visual angle of items was 8° horizontally (334 × 250 pixels) and projected to a screen by using STIM2^TM^ software (Compumedics Neuroscan, Charlotte, NC). Each condition consisted of 200 trials. In addition, 20 trials were included, which contained a perceptual decision period after item presentation wherein the subject responded whether previously displayed item was a building or landscape by pressing a button in order to ensure that the subject attended the item during item presentation. Finally each condition had 220 trials. After the encoding session, the recognition session started after a brief interference task using simple arithmetic calculation. The total 440 items were randomly intermixed with 200 of new items (“New”) as memory foils. The participants were given three buttons, i.e., “old”, “don’t know”, and “new”, and responded by pressing a button while each item was presented on the screen for four seconds.

### Behavioral measurement

All the trials of the recognition session were categorized by the response during the recognition session, as follows. The old and new responses for the trials of R condition were tagged as R-hits and R-misses, respectively. Those for the NR condition were tagged as NR-hits and NR-misses, while those for the new condition as false alarms and correct rejections. To quantify the degree to which a subject followed the instruction depending on a cue sign, instruction compliance was defined by the standard *d*-prime of R-hits minus NR-hits. In addition, memory performance was defined by the standard *d*-prime of R-hits minus false alarms.

### MEG measurement

Brain electromagnetic activities were measured during the encoding session using a whole-head MEG Neuromag (VectorView^TM^, Elekta Neuromag Oy, Helsinki, Finland) acquisition system installed at the MEG center of Seoul National University Hospital. The vertical and horizontal electrooculogram (EOG) and electrocardiogram (ECG) was also recorded to remove eye movements and cardiac artifacts. All subjects entered the electromagnetically shielded and sound-attenuated room after being attached head position indicator (HPI) coils sparsely on the head, and identified anatomical landmarks such as nasion and bilateral preauricular points by 3D digitizer (FASTRAKTM, Polhemus, Colchester, VT). Then HPI coils in the MEG machine registered the subject’s head position, allowing the reconstructed sources of MEG signal to be overlaid on structural MR images with high precision. Before data analysis, a Maxwell filter (Signal Space Separation) was adopted to reduce the possible confounding influence of biological and environmental noises^36^,^37^.

### Structural MRI image acquisition

For the source localization of MEG signal, T1-weighed MR images were acquired by gradient echo pulse sequence (repetition time 1.67 s, echo time 1 ms, and flip angle 9°) at 3 Tesla using a Siemens Trio Tim scanner (Siemens, Erlangen, Germany), yielding 208 sagittal slices with 1.0 × 0.98 × 0.98 mm^3^ voxel size.

### MEG signal preprocessing

All the signal processing from preprocessing to the network analysis was conducted using the Fieldtrip open source software package (the Donders Institute for Brain, Cognition and Behavior, Centre for Cognitive Neuroimaging, Nijmegen, The Netherlands, http://fieldtrip.fcdonders.nl)^38^ and in-house scripts in MATLAB (2013a, MathWorks, Natick, MA, USA). Prior to the spectral analysis, data was downsampled at 600 Hz after applying a low-pass filter at 200 Hz to reduce computational burden. Trials tainted with SQUID jump and muscle artifacts were visually selected to be rejected, and then EOG and ECG artifacts were removed using independent component analysis (ICA).

### Spectral analysis

After artifact removal, time-frequency representations of power were calculated in two distinct frequency ranges. For lower frequencies (1–32 Hz), spectral power was computed based on a sliding time window shifted in steps of 50 ms covering the whole trial length of 4 s. The length of the sliding time window was adapted to the frequency containing four cycles (i.e., ΔT = 4/f, e.g. 400 ms for 10 Hz). Prior to the Fourier transformation, the data segments of the sliding time windows were tapered with a Hanning window, resulting in adaptive spectral smoothing of Δf ~1/ΔT. For high frequency ranges (20–200 Hz), a time window of 200 ms length was applied with a multitaper approach involving three orthogonal Slepian tapers, resulting in a spectral smoothing of ~10 Hz^39^.

### Source analysis

To reconstruct sources in frequency domain, the sources of the oscillatory activities were identified using a beamforming approach based on an adaptive spatial filter (Dynamic Imaging of Coherent Sources, DICS)^40^. We defined two distinct frequency and time windows according to the previous report^7^: 10 Hz during cue presentation (1–2 s, latency 1 s; cue-alpha as described above) and 80 Hz during item presentation (2–3 s, latency 1 s; item-gamma as described above). Subsequently, for Fourier transformation, a multitaper method was used to compute spectrum for the entire segmented data length (1 s). For 10 Hz, a Hanning taper was applied, leading to 3 Hz smoothing for a 500 ms window, while three Slepian tapers for the gamma frequency (80 Hz) resulting in a 10 Hz spectral smoothing. The cross-spectral density matrices were calculated for each Fourier transformed data for each time window, frequency, condition, and subject.

From each individual’s MRI, a realistically shaped single-shell description of the brain was constructed. Each subject’s brain volume was discretized into a grid with a 0.8 cm resolution and spatially normalized to the MNI brain template (International Consortium for Brain Mapping, Montreal Neurological Institute, Canada) by using SPM8 (http://www.fil.ion.ucl.ac.uk/spm). Then, the lead field was computed at every grid point. A spatial filter was formed for each grid point using the cross-spectral density matrices for the frequency of interest and the lead fields. In the end, the spatial distribution of oscillatory power was computed for each condition by applying the common filter for both conditions. The source power spectrum for each cue-alpha and item-gamma was divided by the average of source spectral power and source of ITI period (latency 1 s, 3–4 s) for corresponding spectral band at single trial level. Finally normalized source power for each cue-alpha and item-gamma was displayed in Supplementary Figure 1.

### Modeling the Brain Network

#### Distributed time-delayed cross-frequency network modeling in the form of the bipartite graph

**Figure 6:**
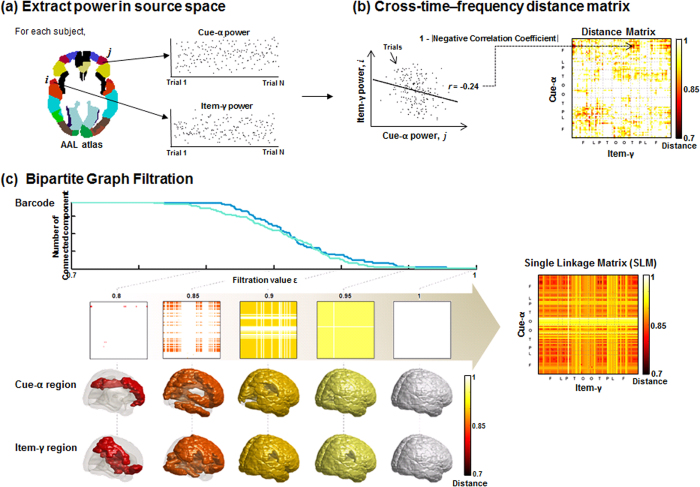
Pipeline of the bipartite graph filtration of the time-delayed cross-frequency network. Network analysis of bipartite graph filtration was performed for each individual and for each Remember and No-Remember condition. The Remember condition of a participant (#1) was used to illustrate the procedure of bipartite graph filtration. (**a**) Nodes were defined by AAL atlas over cerebral cortex omitting subcortical and cerebellar regions. AAL atlas was interpolated into a source grid space, and then source power was extracted to be averaged within each region. (**b**) Power-to-power Pearson’s correlation coefficients were calculated between alpha power during cue presentation and gamma power during item presentation. Correlation coefficients were converted into distance by calculating (1 - absolute values of the negative correlation coefficients). (**c**) Filtering this time-delayed cross-frequency graph, a barcode and a single linkage matrix were produced for each condition of every participant.

To present the relationship between cue-alpha and item-gamma, we modeled a weighted bipartite graph. Nodes were defined by region of interests (ROIs) using the Automated Anatomical Labeling (AAL) atlas^41^ consisting of 38 regions for each hemisphere after excluding subcortical and cerebellar areas (Supplementary Table 1). AAL atlas space was interpolated into an individual source grid space, and then source power values were averaged within each ROI for each cue-alpha and item-gamma per condition and subject (Fig. 6a).

Let the number of ROI called *p*. The cue-alpha measurements is denoted as *X* consisting of 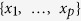, and item-gamma measurement is denoted as *Y* consisting of 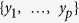. The relationship between *x*_*i*_ at *i-*th node and *y*_*j*_at *j-*th node is defined by the Pearson’s correlation coefficient 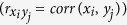, and its distance 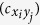 is as follows.


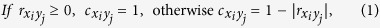


where each *i* and *j* is composed of 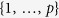, and the range of 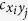 can be from 0 to 1.

The correlation was computed across trials, while the number of trials between two task conditions (R and NR) was slightly different for each subject due to trial rejection for artifact removal. Considering that correlation coefficients are affected by the number of observations (i.e., trials)^42^, the number of trials was adjusted to the minimum number of trials between two conditions by collecting trials randomly for each subject before calculating Pearson’s correlation coefficients (Fig. 6b). According to the findings of the previous study^7^, the decreased power of cue-alpha was supposed to represent the opening of a gate to the memory system, which was related to the increased power of item-gamma. Whereas, increased power of cue-alpha indicated blocking the ensuing encoding process, which was represented by decreased power of item-gamma. As pre-stimulus regulation over the following encoding process has been suggested to be operated by negative relationship between cue-alpha power and item-gamma power, we took into account only the negative relationship for further analysis in this study. Thus we assigned the maximum distance (i.e., 1) to 
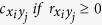
.

#### Bipartite graph filtration for time-delayed cross-frequency network

First, we define a constant, ε, to confine edges weights of a network to be drawn. The thresholded or unweighted network is constructed by connecting two nodes *x*_*i*_ *and y*_*j*_ are connected with an edge if the distance, 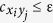. As the ε goes from 0 to 1, a nested sequence of the thresholded networks is formed. This procedure is called graph filtration and the ε is called a filtration value. The present study focused only on the number of connected components, Betti-0 as the topological feature of the thresholded network and visualized it by plotting the birth and the death intervals of connected components. The topological feature of the network could be visualized in two ways: the barcode and dendrogram^8^. Dendrogram showed the hierarchically clustered structure of a graph while the nodes are fixed along the y axis unlike the barcode where the nodes are shuffled.

While we applied graph filtration to the bipartite graph of time-delayed cross-frequency alpha-gamma coupling, yielding the bipartite graph filtration (Fig. 6c) and the resulting filtered bipartite networks were presented using two disjoint sets of nodes of cue-alpha and item-gamma of Remember or No-Remember condition, during cue and item presentations, respectively. In the meantime, we filtered graphs of inter-regional correlation in the same frequency band and time, namely between cue-alpha and cue-alpha, and between item-gamma and item-gamma in their distributed spatial negative relationships. Then we compared the resulting SLMs and barcodes with those of bipartite filtered graph. The comparison of bipartite filtered graph for time-delayed cross-frequency with non-time-delayed within-frequency network for cue-alpha was supposed to find the effect of bipartite connections (or possibly compared with non-time-delayed within-frequency network for cue gamma but we skipped this analysis as we attended to alpha spectral power of cue presentation). In the same way, we compared bipartite filtered graph for time-delayed cross-frequency with non-time-delayed within-frequency network for item-gamma. These comparisons would disclose the contribution of disjoint bipartite connections in the distributed time-delayed cross-frequency coupling by removing the contribution of non-time-delayed within-frequency coupling.

The barcode and dendrogram could examine only the global differences between R and NR condition. For examination of the local difference in the connected structure of network, we computed SLM as well. The SLM is derived based on the study^8^, which is defined by


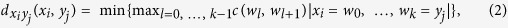


where 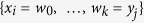 is a route between *x*_*i*_ and *y_j_*. The *d* indicates the minimum step to reach each other node, which can be simplified as 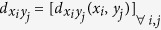 representing the sequence of formation of the clusters, or connected component. In the end, the SLM as well as dendrogram were served as findings of topological invariants that quantify the hierarchical connected structure of the network. For better understanding of the bipartite graph filtration, we provided an example of bipartite graph and MATLAB codes at https://sites.google.com/site/jaranghahm/home/bipartite-graph-filtration.

All the bipartite graph filtration and further analyses were done in individuals and the filtered bipartite graphs resulting barcodes, dendrograms and SLMs were grouped together according to the memory performance. The 23 subjects were divided into good (standard *d*-prime of R-hits minus false alarms >1.0) and poor (<1.0) performance groups. The results of good performers and poor performers were compared with each other in *d*_*ij*_ of SLMs and barcodes. Which regional pair of time-delayed cross-frequency network was predictive of memory performance was looked for by correlation analysis between *d*_*ij*_ and instruction compliance, and between *d*_*ij*_ and memory performance.

### Statistical analysis

#### Comparison of the Barcode

The global difference was tested by analyzing barcodes. Time-delayed cross-frequency network did not allow the generation of barcodes, so we separately computed the barcode according to each set of nodes. For the barcode of time-delayed cross-frequency filtered graph for cue-alpha, it was compared with the non-time-delayed within-frequency network for cue-alpha. The difference in the barcode between the cue-alpha of the time-delayed cross-frequency network and the cue-alpha of the non-time-delayed within-frequency filtered network was defined by the difference in the number of connected components (*β*_0_) whose magnitude of difference was the maximum across filtration values, and we called it diff_max_. In computation, we found 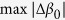 between two barcodes across the filtration values and the corresponding 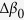 became a diff_max_. The significance of diff_max_ between time-delayed cross-frequency network and non-time-delayed within-frequency network for each R and NR conditions was tested using Wilcoxon signed rank test for each the graph for cue-alpha and item-gamma.

**Figure 7:**
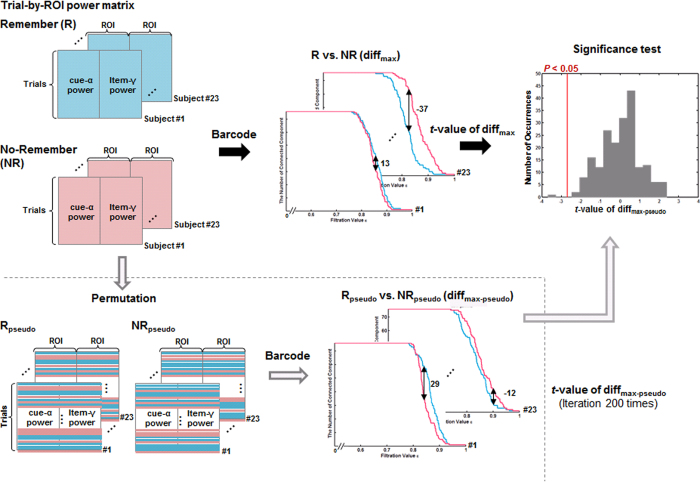
Comparison of the barcode using the permutation test. To compare the barcodes between conditions for each cue-alpha and item-gamma, we used the maximum difference of the barcode (diff_max_) defined by the value whose absolute value showed the maximum difference between two barcodes across filtration values. The diff_max_ was computed for each subject and each condition. The diff_max_ was tested based on the permutation test, and we chose the one-sample *t* statistic as the permutation statistic. For building a null distribution, the trials of all the Remember (R) and No-Remember (NR) conditions were randomly re-assigned into two groups, yielding two pseudo conditions, R_pseudo_ and NR_pseudo_ condition. For this pseudo condition data, the randomized *t* statistic was calculated. By iterating this procedure 200 times, 200 randomized *t* statistic values built a null distribution. The *t* statistic observed from the original R and NR data was located on the null distribution and its *P-*value was computed. The figure illustrated this procedure, and the distribution on the right side showed the results of item-gamma: the significance was *P* = 0.01.

Subsequently, barcodes of the R condition were compared with those of the NR condition and its significance was tested using the permutation test (Fig. 7). In individuals, the R condition was compared with the NR condition in barcodes of each cue-alpha and item-gamma of the time-delayed cross-frequency network. The difference of barcodes between conditions was represented by diff_max_ and tested against the null distribution acquired from the randomly assigned of R and NR conditions at trial level. To examine whether differences in the barcode between conditions is related to instruction compliance, the correlation coefficient was calculated between the diff_max_ and instruction compliance (the standard *d*-prime of R-hits minus NR-hits).

#### Comparison of the Single Linkage Matrix

**Figure 8:**
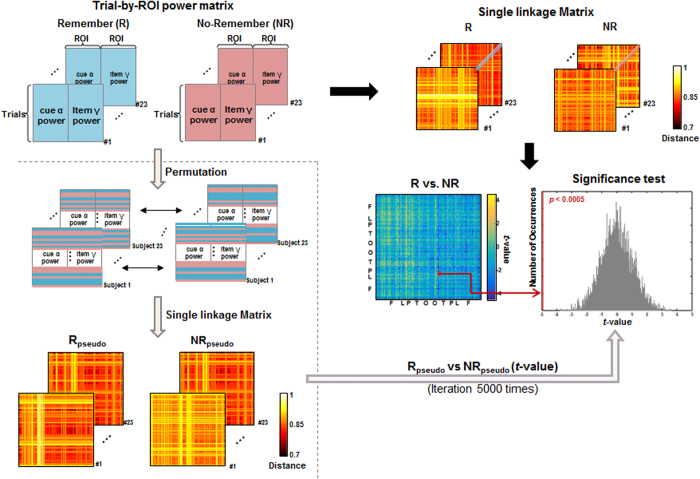
Comparison of the single linkage distance using the permutation test. The paired version of *t* statistic was used to compare Remember and No-Remember condition at every single linkage distance in the single linkage matrix 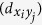. The significance of differences of 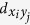 between conditions was tested using the permutation test. All the trials of both R and NR conditions were randomly re-assigned into two groups, yielding two pseudo conditions, R_pseudo_ and NR_pseudo_. For pseudo conditions, single linkage distance was computed and calculated the *t*-statistic. This procedure iterated 5,000 times and 5,000 *t* statistics generated a null distribution. The *P* -values of the observed *t* statistic of every 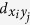 were computed against the null distribution.

To find specific connectivity showing difference between R and NR condition, we tested statistical differences of SLMs 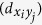 between conditions by a non-parametric method using the *t*-statistic across subjects (mean 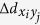 between conditions over square root (sample variance of 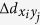/sample size)) as variables. A null hypothesis was that two conditions of R and NR were not different, so that the trials of two conditions were exchanged randomly within individual subjects to reassign R condition with NR condition and vice versa. The pseudo-conditions of R and NR made of 5,000 times of reassigned trials in each individual yielded a null distribution of difference of SLMs between conditions. Finally the significance of difference in 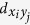 of SLM between two conditions was tested at *P* < 0.0005 based on this null distribution (Fig. 8). For further analysis, we chose regional pairs with *P*-value less than 0.0005 for either ‘R > NR’ or ‘R < NR.’ Once any regional pair was found to differ between R and NR condition in their single linkage distances, we examined whether 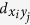 of regional pair was related to instruction compliance (the standard *d*-prime of R-hits minus NR-hits) or with memory performance (the standard *d*-prime of R-hits minus false alarms). Again, the regional pair of ‘R < NR’ was supposed to mean the encoding network according to the instruction of R, while the regional pair of ‘R > NR’ was supposed to mean the distraction network to obey task instruction of NR.

After grouping the entire subjects to good performers and poor performers according to memory performance (the standard *d*-prime of R-hits minus false alarms), we grouped the subjects to pseudo-groups by randomizing the assignment of the subjects to pseudo-good performers and pseudo-poor performers 5,000 times. From these pseudo groups, we performed bipartite graph filtration to make SLMs of pseudo-good performers and pseudo-poor performers. Observed differences of 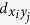 of SLMs 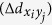 of good and poor performers were tested for their significance against the null distribution of 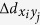 from pseudo-groups. For further analysis, we chose regional pair with *P-*value less than 0.0005. Once regional pairs were found to have different single linkage distances between good performers and poor performers, we related 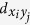 of SLMs of time-delayed cross-frequency networks of the individuals with their memory performance (the standard *d*-prime of R-hits minus false alarms).

## Additional Information

**How to cite this article**: Hahm, J. *et al*. Gating of memory encoding of time-delayed cross-frequency MEG networks revealed by graph filtration based on persistent homology. *Sci. Rep.* **7**, 41592; doi: 10.1038/srep41592 (2017).

**Publisher's note:** Springer Nature remains neutral with regard to jurisdictional claims in published maps and institutional affiliations.

## Supplementary Material

Supplementary Figure and Table

## Figures and Tables

**Table 1:** The significant single linkage distance between Remember and No-Remember condition (*P* < 0.0005, uncorrected for multiple comparisons).

Connectivity		Mean	SE	Range	*t*-value	Correlation with compliance	Correlation with memory performance
Cue presentation	Item presentation						
Superior occipital gyrus (L)	Superior frontal gyrus, dorsolateral (L)	−0.035	0.008	−0.094–0.047	−4.195	−0.576*	−0.428
Middle Temporal gyrus (R)	Middle frontal gyrus, orbital part (L)	−0.031	0.007	−0.084–0.051	−4.598	−0.252	−0.113
Inferior frontal gyrus, opercular part (R)	Insula (L)	−0.027	0.007	−0.077–0.035	−3.902	−0.427	−0.404
Cunues (L)	Insula (L)	−0.038	0.009	−0.127–0.074	−4.072	−0.410	−0.348
Temporal pole: superior temporal gyrus (L)	Insula (L)	−0.030	0.007	−0.087–0.051	−4.569	−0.243	−0.174
Supramarginal gyrus (L)	Insula (L)	−0.040	0.008	−0.090–0.051	−4.923	−0.192	−0.155
Superior frontal gyrus, orbital part (L)	Insula (L)	−0.039	0.008	−0.100–0.032	−4.888	−0.314	−0.226

^*^P < 0.05 for Bonferroni correction on correlation with behavioral measures. SE: Standard error, L: Left, R: Right.
