# Supplementary material for: Gating of memory encoding of time-delayed cross-frequency MEG networks revealed by graph filtration based on persistent homology
Source: Sci Rep. 2017 Feb 7;7:41592. doi: 10.1038/srep41592 (PMC5294648; doi:10.1038/srep41592)
Supplement: Supplementary Figure and Table [file srep41592-s1.pdf]

Gating of memory encoding of time-delayed cross-frequency MEG networks revealed by graph filtration based on persistent homology

Jarang Hahm[1, 2, 3], Hyekyoung Lee[1, 2], Hyojin Park[1, 2, 3], Eunjoo Kang[4], Yu Kyeong Kim[1, 6, 8], *Chun Kee Chung[5], *Hyejin Kang[1, 6], *Dong Soo Lee[1, 2, 3, 6, 7]

(*asterisk indicated a corresponding author)

[1]Department of Nuclear Medicine, Seoul National University College of Medicine, Seoul, 110-744, Korea,

[2]Institute of Radiation Medicine, Medical Research Center, Seoul National University, Seoul, 110-744, Korea

[3]Interdisciplinary Program in Cognitive Science, Seoul National University, Seoul, 151-742, Korea

[4]Department of Psychology, Kangwon National University, Chuncheon-si, 200-701, Korea

[5]MEG Center, Department of Neurosurgery, Seoul National University College of Medicine, Seoul, 110-744, Korea

[6]Data Science for Knowledge Creation Research Center, Seoul National University, Seoul, 151-742, Korea

[7]Department of Molecular Medicine and Biopharmaceutical Sciences, Graduate School of Convergence Science and Technology, and College of Medicine or College of Pharmacy, Seoul National University, Seoul, 110-744, Korea

[8]Department of Nuclear Medicine, Seoul National University Boramae Medical Center, 156-707, Seoul, Korea

Supplementary Figure 1. Comparison of the source spectral power after normalization at single trial level.

To model a functional brain network for each subject and condition, source spectral power was normalized at single trial level. Grand average (N=23) of differences between R and NR conditions after power normalization were shown. Alpha power was lower during cue presentation and gamma power was stronger during item presentation in R than NR condition, especially in the posterior region.

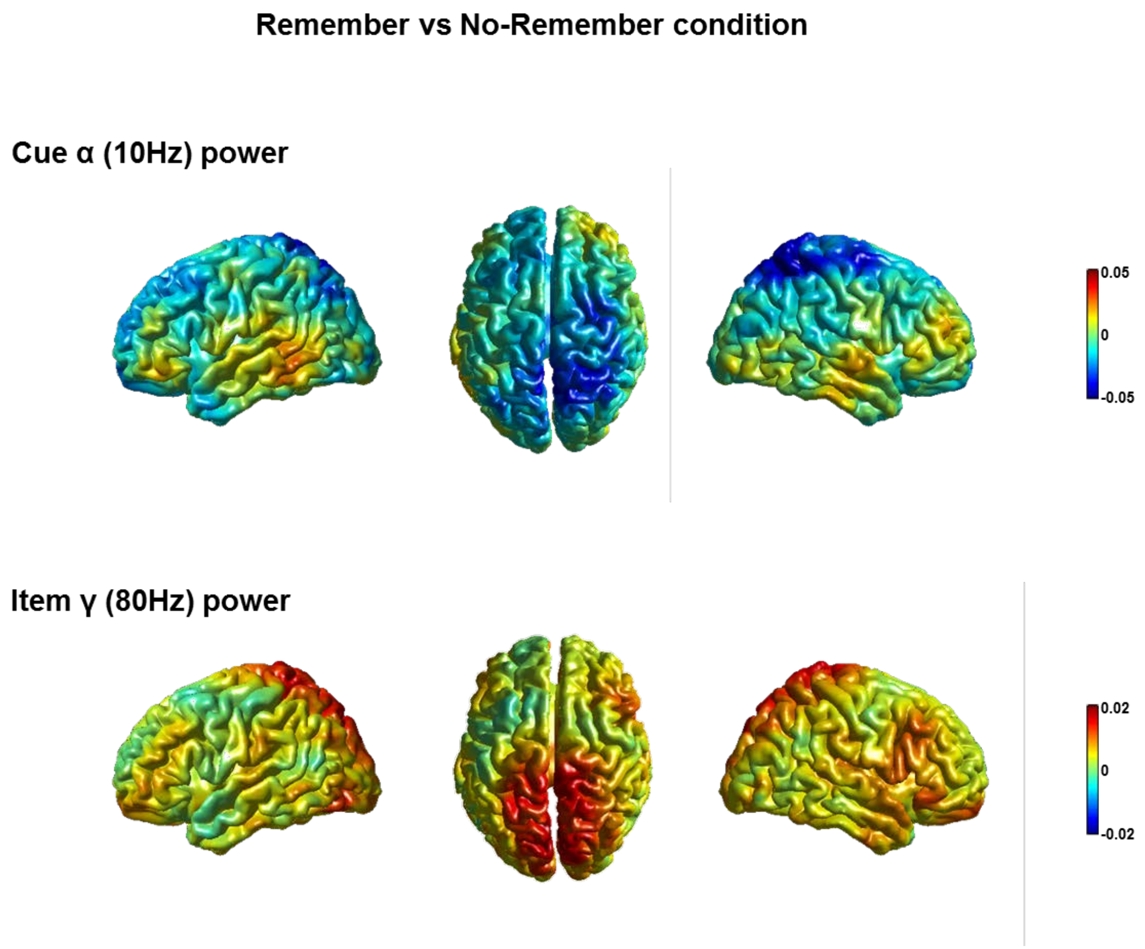

Supplementary Table 1. The list of region of interests (ROIs) used for network analysis. Total 38 regions for each hemisphere were selected except for subcortical and cerebellar regions.

**No.**	**Regional category**	**Anatomical description**	**Abbreviation**
1	Frontal	Olfactory cortex	OFC
2		Gyrus rectus	Rectus
3		Inferior frontal gyrus, orbital part	IFG.orb
4		Inferior frontal gyrus, opercular part	IFG.oper
5		Inferior frontal gyrus, triangular part	IFG.tri
6		Middle frontal gyrus, orbital part	MFG.orb
7		Middle frontal gyrus	MFG
8		Superior frontal gyrus, orbital part	SFG.orb
9		Superior frontal gyrus, medial orbital	MFG.orb
10		Superior frontal gyrus, medial	SFG.med
11		Superior frontal gyrus, dorsolateral	SFG.dl
12		Paracentral lobule	Paracentral
13		Supplementary motor area	SMA
14		Precentral gyrus	Precentral
15		Rolandic operculum	Rolandic.oper
16	Limbic	Anterior cingulate and paracingulate gyri	Cing.ant
17		Median cingulate and paracingulate gyri	Cing.mid
18		Posterior cingulate gyrus	Cing.post
19	Parietal	Postcentral gyrus	Postcentral
20		Superior parietal gyrus	SPC
21		Precuneus	PreCu

22		Inferior parietal, but supramarginal and angular gyri	IPC
23		Supramarginal gyrus	SMG
24		Angular gyrus	AG
25	Temporal	Superior temporal gyrus	STG
26		Heschl gyrus	Heschl
27		Middle temporal gyrus	MTG
28		Inferior temporal gyrus	ITG
29		Temporal pole: superior temporal gyrus	TP.STG
30		Temporal pole: middle temporal gyrus	TP.MTG
31		Insula	INS
32	Occipital	Fusiform gyrus	Fusi
33		Inferior occipital gyrus	IOC
34		Middle occipital gyrus	MOC
35		Superior occipital gyrus	SOC
36		Calcarine fissure and surrounding cortex	Calcarine
37		Cuneus	Cu
38		Lingual gyrus	Ling
